# Nutrient-dependent control of RNA polymerase II elongation rate regulates specific gene expression programs by alternative polyadenylation

**DOI:** 10.1101/gad.337212.120

**Published:** 2020-07-01

**Authors:** Carlo Yague-Sanz, Yann Vanrobaeys, Ronan Fernandez, Maxime Duval, Marc Larochelle, Jude Beaudoin, Julien Berro, Simon Labbé, Pierre-Étienne Jacques, François Bachand

**Affiliations:** 1 Department of Biochemistry and Functional Genomics, Université de Sherbrooke, Sherbrooke, Québec J1E 4K8, Canada;; 2 Department of Molecular Biophysics and Biochemistry, Yale School of Medicine, New Haven, Connecticut 06520, USA;; 3 Department of Cell Biology, Yale School of Medicine, New Haven, Connecticut 06520, USA;; 4 Department of Biology, Université de Sherbrooke, Sherbrooke, Québec J1K 2R1, Canada

**Keywords:** transcription elongation rate, alternative polyadenylation, RNA polymerase II, phosphate starvation, transcription termination, NTP sensing

## Abstract

Transcription by RNA polymerase II (RNAPII) is a dynamic process with frequent variations in the elongation rate. However, the physiological relevance of variations in RNAPII elongation kinetics has remained unclear. Here we show in yeast that a RNAPII mutant that reduces the transcription elongation rate causes widespread changes in alternative polyadenylation (APA). We unveil two mechanisms by which APA affects gene expression in the slow mutant: 3′ UTR shortening and gene derepression by premature transcription termination of upstream interfering noncoding RNAs. Strikingly, the genes affected by these mechanisms are enriched for functions involved in phosphate uptake and purine synthesis, processes essential for maintenance of the intracellular nucleotide pool. As nucleotide concentration regulates transcription elongation, our findings argue that RNAPII is a sensor of nucleotide availability and that genes important for nucleotide pool maintenance have adopted regulatory mechanisms responsive to reduced rates of transcription elongation.

## Abstract (precis)

In this study from Yague-Sanz et al., the authors investigated the physiological relevance of variations in RNAPII elongation kinetics, and show in yeast that a RNAPII mutant that reduces the transcription elongation rate causes widespread changes in alternative polyadenylation (APA). Their findings indicate that RNAPII is a sensor of nucleotide availability and that genes important for nucleotide pool maintenance have adopted regulatory mechanisms responsive to reduced rates of transcription elongation.

Transcription of eukaryotic genes by RNA polymerase II (RNAPII) is a fundamental process composed of three sequential, yet interconnected steps that consist of initiation, elongation, and termination. During this transcription cycle, transcription units are generally characterized by a conserved 5′-to-3′ profile of phosphorylation on the carboxy-terminal domain (CTD) of Rpb1, the catalytic subunit of RNAPII. Rpb1 CTD heptad repeats, which consist of the consensus amino acid sequence Y_1_-S_2_-P_3_-T_4_-S_5_-P_6_-S_7_, undergo dynamic changes in phosphorylation during transcription elongation, such that Ser5 phosphorylation (Ser5P) typically peaks at the 5′ end, whereas Ser2P is highest at the 3′ end (Zaborowska et al. 2016; Harlen and Churchman 2017). These CTD phosphorylation and dephosphorylation cycles during the elongation phase are key to coordinate the sequential recruitment of RNA processing factors along transcription units. For instance, at the 3′ end of genes, Ser2P contributes to the cotranscriptional recruitment of conserved 3′ end processing factors such as Pcf11 and Seb1, which, together with cleavage and polyadenylation factors, promote endonucleolytic RNA cleavage and polyadenylation [poly(A)] site selection (Meinhart and Cramer 2004; Lunde et al. 2010; Lemay et al. 2016; Wittmann et al. 2017). In addition to releasing the nascent transcript for polyadenylation, the cleavage mediated by the 3′ end processing machinery also provides an uncapped 5′ entry point for a conserved protein complex that includes the 5′–3′ exonuclease Rat1/Xrn2 (Kim et al. 2004; West et al. 2004). This complex is thought to promote transcription termination by chasing RNAPII and ultimately triggering RNAPII dissociation along the chromatin template (Porrua et al. 2016; Proudfoot 2016). Termination of RNAPII transcription thus requires cleavage at the poly(A) site followed by cotranscriptional degradation of the downstream nascent transcript.

Transcription elongation is normally viewed as a continuous process during which RNAPII traverses the coding region after initiating at a promoter. However, genome-wide studies indicate that transcription elongation by RNAPII is a rather intermittent process, with frequent occurrences of pausing, backtracking, and arrest (Churchman and Weissman 2011; Gómez-Herreros et al. 2012; Sheridan et al. 2019). Furthermore, rates of transcription elongation in coding regions appear to differ between genes and within transcriptional units (Danko et al. 2013; Jonkers et al. 2014; Veloso et al. 2014; Cortazar et al. 2019). As several key steps of RNA maturation occur cotranscriptionally, variations in elongation rates are therefore expected to have important consequences on the efficiency of pre-mRNA processing. For example, using Rpb1 catalytic mutants that transcribe faster or slower than normal, it has been shown that changes in the transcription elongation rate can affect the outcome of pre-mRNA splicing in both yeast and human cells (Roberts et al. 1998; de la Mata et al. 2003; Fong et al. 2014; Aslanzadeh et al. 2018). Conversely, pre-mRNA splicing also appears to affect transcription elongation rates (Kornblihtt et al. 2004; Alexander et al. 2010), suggesting that both processes mutually contribute to promote efficient and accurate coupling between transcription and RNA processing. Current models therefore suggest that regulation of RNAPII elongation kinetics can influence splicing such that slowing down the rate of transcription elongation increases the opportunity of an upstream splice site to be recognized before a competing downstream splice site is transcribed (Bentley 2014).

Another cotranscriptional process potentially controlled by RNAPII elongation rate is poly(A) site selection in alternative polyadenylation (APA). APA occurs when genes harbor multiple poly(A) sites, which give rise to mRNA isoforms with 3′ untranslated regions (UTRs) of different lengths (Tian and Manley 2017). Accordingly, it has been proposed that faster elongation rates would increase the competition between proximal and distal poly(A) sites, whereas a slow transcribing RNAPII would favor usage of proximal poly(A) sites (Pinto et al. 2011; Liu et al. 2017a). APA plays important roles in posttranscriptional gene regulation, as 3′ UTR elements contain sequence motifs that can regulate gene expression via RNA stability, translation control, and RNA localization (Mayr 2017).

Although evidence strongly supports the view that the rate of RNAPII elongation can influence cotranscriptional RNA processing, how kinetics of RNAPII passage through chromatin globally affects gene expression remains poorly understood. Furthermore, whether variations in transcription elongation rate are used in a natural context to control the expression of a specific set of genes is largely unknown. In this work, we analyzed the genome-wide expression changes imposed by a Rpb1 catalytic mutant with a reduced elongation rate in fission yeast. We found that a conserved core regulon sensitive to phosphate starvation (PHO regulon) is activated by a slowly transcribing RNAPII via a mechanism that involves APA and premature transcription termination of interfering upstream long noncoding (lnc) RNAs. Importantly, we show that APA and premature termination are naturally used to regulate this core PHO regulon upon phosphate starvation. Collectively, our data reveal that a reduction in the transcription elongation rate causes a distal-to-proximal shift in APA, resulting in increased expression of a specific set of genes that includes the core PHO regulon and genes involved in the purine biosynthesis pathway. Our findings support a model in which phosphate limitation results in reduced elongation rates as a consequence of nucleotide depletion and that phosphate-responsive genes have evolved activation mechanisms sensitive to suboptimal transcription elongation kinetics.

## Results

### A mutant of RNA polymerase II with a reduced elongation rate causes specific changes in gene expression

**Figure 1.**
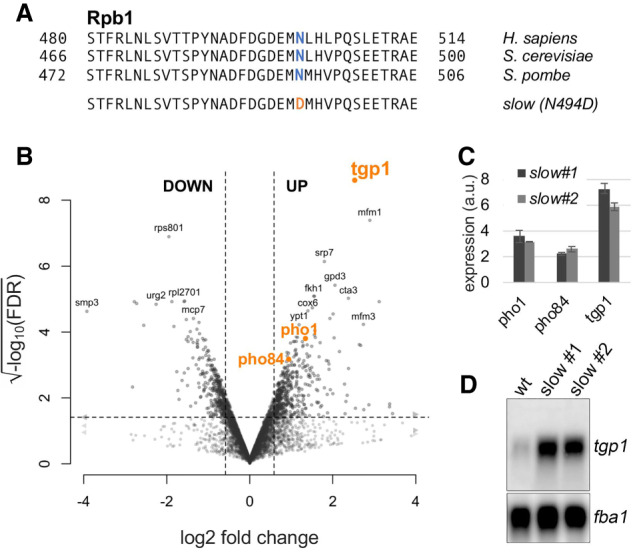
Transcriptome-wide analysis of gene expression changes in the *S. pombe* Rpb1 slow mutant. (*A*) Multiple sequence alignment of a conserved region in the catalytic core of Rpb1. The conserved asparagine residue is colored in blue, whereas the substitution is colored in orange in the slow mutant. (*B*) Volcano plot of statistical significance against fold change (in log_2_) of gene expression in the *slow* mutant relative to the wild-type control (*n* = 7049 genes). The dashed lines represent the thresholds for calling significant differential gene expression: FDR <0.01 and absolute log_2_ fold change >log_2_(1.5). To ease viewing, genes with values beyond axes limits are represented by arrowheads. The phosphate-responsive genes *tgp1*, *pho1*, and *pho84* are highlighted in orange. (*C*) RT-qPCR analysis of *tgp1*, *pho1*, and *pho84* mRNA levels in independent slow mutants relative to the wild-type parental strain from three independent experiments. (a.u.) Arbitrary units. Error bars represent the standard deviation of the mean. (*D*) Validation of *tgp1* up-regulation in independent Rpb1 slow mutants by Northern blot. The *fba1* mRNA was used as a loading control.

To investigate how a slow transcription elongation rate globally affects gene expression, we introduced a mutation that was originally identified in a genetic screen for hypersensitivity to the nucleotide-depleting drug 6-azauracil (6AU) in *S. cerevisiae* (Malagon et al. 2006) in the gene encoding *S. pombe rpb1*, the largest subunit of RNA polymerase II (RNAPII). This mutation substitutes an evolutionarily conserved asparagine at position 494 of *S. pombe* Rpb1 to an aspartic acid residue (N494D) (see Fig. 1A) and corresponds to the N488D substitution in *S. cerevisiae* Rpb1 that results in reduced elongation rates in vitro (Malagon et al. 2006) and in vivo (Jimeno-González et al. 2010) as well as in increased transcriptional slippage (Strathern et al. 2013). Asparagine 494 is located below the active site in the Rpb1 catalytic loop (see Supplemental Fig. S1), in the vicinity of the magnesium ions and the incoming nucleoside triphosphates (Strathern et al. 2013). To generate a mutant strain that expresses Rpb1 N494D from the endogenous *rpb1* locus, we used CRISPR/Cas9-based genome editing (Fernandez and Berro 2016) and isolated independent clones that displayed the desired *rpb1* mutation after DNA sequencing. Importantly, the *rpb1-N494D* mutant was sensitive to 6AU and mycophenolic acid (MPA) (Supplemental Fig. S2), drugs known to deplete intracellular NTP pools and stress transcription elongation, as previously shown for the analogous *S. cerevisiae* mutant (Malagon et al. 2006).

To measure the impact of the RNAPII slow mutant on gene expression, we analyzed total RNA prepared from two independent *rpb1-N494D* clones as well as the parental control strain by strand-specific RNA-seq. Expression data from the two N494D mutants clustered together (Supplemental Fig. S3A), showing strong reproducibility between the independent clones. In total, 323 genes were down-regulated and 457 were up-regulated in the slow mutant (Fig. 1B; Supplemental Table S1). To further validate our RNA-seq experiment, we measured the change of expression of 10 differentially expressed genes by RT-qPCR and observed good overall correlation (*R*^2^= 0.92) with the RNA-seq data (Supplemental Fig. S3B,C). Down-regulated genes were enriched for gene ontology categories related to transmembrane transport, ribosome biogenesis, and iron assimilation (Supplemental Fig. S4A), whereas up-regulated genes were significantly enriched for genes involved in purine (especially ATP) biosynthesis as well as sexual reproduction (Supplemental Fig. S4B). Many of the up-regulated genes in the slow mutant are direct targets of Ste11, which is induced in the slow mutant and functions as the master transcription factor for sexual development in fission yeast. Thus, up-regulation of genes such as *mei2*, *mfm1*, *mfm2*, and *mfm3* is likely to be an indirect consequence of *ste11* induction.

We also examined for splicing deficiencies in the *rpb1* slow mutant, as previous studies indicate that RNAPII elongation rate can influence splicing efficiency (de la Mata et al. 2003; Fong et al. 2014; Aslanzadeh et al. 2018). Two independent computational approaches were used on our RNA-seq data to examine the impact of RNAPII slowdown on splicing efficiency (Li et al. 2020). However, none of these analyses supported strong global changes in RNA splicing in the *rpb1* slow mutant (Supplemental Fig. S5). Moreover, intron-containing genes were neither enriched in the set of genes that were significantly up-regulated nor in the set of genes significantly down-regulated in the slow mutant. We therefore conclude that the *rpb1 N494D* slow mutant affects the expression of a specific set of genes but does not largely influence RNA splicing.

### A conserved regulon that responds to phosphate starvation is induced in the RNAPII slow mutant

Fission and budding yeasts share a core regulon responsible for the initial response to phosphate limitations comprising the *pho1*, *pho84*, and *tgp1* genes, which encode phosphate harvesting and transport proteins (Carter-O'Connell et al. 2012). Notably, all three genes of the core PHO regulon were significantly up-regulated in the RNAPII slow mutant (Fig. 1B); yet, expression from those genes should have been repressed as cells were grown in phosphate-containing media. These results were validated by RT-qPCR for the *pho1*, *pho84*, and *tgp1* genes (Fig. 1C) as well as by Northern blot analysis for the *tgp1* mRNA (Fig. 1D). These data reveal that expression of the conserved core PHO regulon is particularly sensitive to a reduction in the transcription elongation rate.

### Premature termination of *nc-tgp1* lncRNA transcription allows for *tgp1* induction in the RNAPII slow mutant

**Figure 2.**
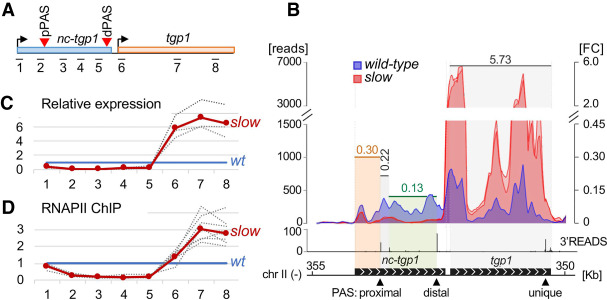
Premature termination of *nc-tgp1* transcription in the RNAPII slow mutant. (*A*) On-scale representation of the *nc-tgp1/tgp1* locus indicating the position of the amplicons (1–8) used for ChIP and qRT-PCR analyses throughout this study as well as the position of the proximal (pPAS) and distal (dPAS) poly(A) sites in *nc-tgp1.* (*B*) Normalized read coverage (*left* axes) at the *nc-tgp1/tgp1* locus for the wild-type (blue area) and slow mutants (red areas) based from the RNA-seq data, and for previously published 3′ READS data (Liu et al. 2017b) in wild-type cells (black peaks at the *bottom*). The fold change (FC; *right* axis) is calculated between averaged mutant versus wild-type values for gene segments delimited by 3′READS peaks, which are represented by shaded vertical areas. (*C*) RT-qPCR analysis of *nc-tgp1/tgp1* expression relative to wild-type cells in three independent experiments. The blue and red lines correspond to the baseline wild-type level and the mean value for the slow mutant, respectively. The individual replicates are shown as dashed lines. (*D*) Relative RNAPII occupancy in the slow mutant compared with the wild type at the *nc-tgp1/tgp1* locus from nine independent experiments. The blue and red lines correspond to the baseline wild-type level and the mean value, respectively, for the slow mutant. The individual replicates are shown as dashed lines.

**Figure 3.**
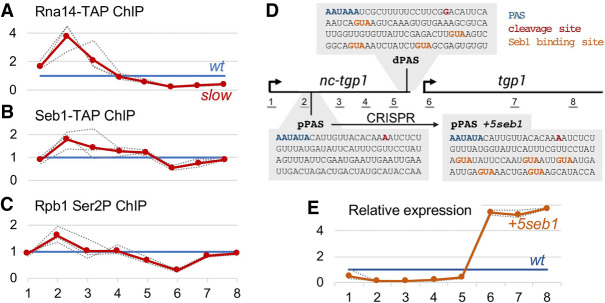
Redistribution of 3′ end processing factors and CTD Ser2 phosphorylation to the proximal poly(A) site of *nc-tgp1* in the RNAPII slow mutant. (*A*–*C*) Chromatin occupancy of Rna14 (*A*), Seb1 (*B*), and Rpb1 CTD Ser2 phosphorylation (*C*) at the *nc-tgp1/tgp1* locus in the RNAPII slow mutant (red line) relative to the wild-type control (blue line). Individual replicates (*n* = 3; dashed lines) were normalized to total RNAPII levels measured from the same chromatin preparation at each region and expressed relative to the wild type. The positions of the amplicons (1–8) used throughout this study are shown in Figure 3D. (*D*) On-scale representation of the *nc-tgp1/tgp1* locus indicating the nucleotide sequences surrounding the proximal and distal poly(A) sites (pPAS and dPAS, respectively) of *nc-tgp1*. The modifications introducing five Seb1-binding sites downstream from the proximal *nc-tgp1* (pPAS +*5seb1* mutant) by CRISPR are shown at the *right*. (*E*) RT-qPCR analysis of *nc-tgp1/tgp1* expression relative to wild type from three independent +*5seb1* clones. The blue and orange lines correspond to the baseline wild-type level and the mean value for the +*5seb1* mutant, respectively. The individual replicates are shown as dashed lines.

To begin to elucidate the molecular mechanisms by which the slow RNAPII mutant results in gene activation, we focused on *tgp1*, which is the most highly induced gene in the Rpb1 slow mutant based on the RNA-seq data. *tgp1* expression has been previously studied in fission yeast (Ard et al. 2014; Ard and Allshire 2016; Sanchez et al. 2018a) and shown to be negatively regulated by a long noncoding (lnc) RNA expressed from the upstream *nc-tgp1* locus (Fig. 2A). However, the exact mechanism by which the repressive effect of *nc-tgp1* transcription is disabled to allow *tgp1* activation upon phosphate starvation has been elusive. Interestingly, our transcriptome-wide analysis of poly(A) site (PAS) mapping in *S. pombe* using 3′READS (Liu et al. 2017b) indicated the presence of at least two PASs in the *nc-tgp1* locus: a minor proximal PAS and a major distal PAS, the latter being surrounded by strong consensus motifs (Figs. 2A,B, 3D). Notably, by comparing the RNA-seq coverage in *nc-tgp1* and *tgp1* between the wild-type strain and the slow mutant, we found that whereas *tgp1* mRNA levels were upregulated in the RNAPII slow mutant (Fig. 2B, see red over blue signal), the levels of the upstream *nc-tgp1* lncRNA were strongly repressed in the slow mutant (Fig. 2B, blue over red signal). This observation was validated by RT-qPCR analysis using primer pairs positioned along the *nc-tgp1* and *tgp1* loci (see Fig. 2A), confirming the reduction of *nc-tgp1* lncRNA concomitant with increased *tgp1* mRNA levels in the slow mutant (Fig. 2C). Similar observations were seen for the other two core PHO regulon genes, *pho84* and *pho1* (Supplemental Fig. S6A). Reduction of the *nc-tgp1* lncRNA in the RNAPII slow mutant was also confirmed by RNase protection assays (Supplemental Fig. S6B). Importantly, by segmenting the *nc-tgp1* locus into different regions based on PAS identification (Liu et al. 2017b), we noted a 5′-to-3′ decrease in the RNA-seq signal ratio in the mutant relative to the wild-type strain (Fig. 2B), suggesting a reduction in the production of the full-length *nc-tgp1* lncRNA. One mechanism by which the RNAPII slow mutant could result in decreased production of full-length *nc-tgp1* is by increased usage of the proximal PAS. As transcription termination is intimately coupled to RNA 3′ end processing (Porrua et al. 2016; Proudfoot 2016), this would be expected to trigger premature termination. To test this hypothesis, we analyzed RNAPII occupancy by chromatin immunoprecipitation (ChIP) assays. As shown in Figure 2D, the Rpb1 slow mutant demonstrated increased RNAPII density in the *tgp1* locus relative to the control strain (see regions 6–8), consistent with up-regulation of *tgp1* mRNA in *rpb1-N494D* cells. In contrast, RNAPII levels markedly declined over the proximal *nc-tgp1* poly(A) site in the slow mutant (regions 2–5), consistent with the idea that the slower elongation rate in *rpb1-N494D* cells promotes premature transcription termination at the *nc-tgp1* locus. Our data argue that the induction of *tgp1* expression in the RNAPII slow mutant results from premature termination of *nc-tgp1* transcription.

### Enrichment of 3′ end processing factors at the proximal nc-tgp1 PAS in the RNAPII slow mutant

ChIP-seq analyses of components of the RNA 3′ end processing machinery show enrichment at the 3′ end of genes (Larochelle et al. 2018) where they promote cotranscriptional RNA cleavage and transcription termination. To measure the effect of the RNAPII slow mutant on the recruitment of the 3′ end processing machinery at the *nc-tgp1* locus, we used ChIP analysis to monitor the binding profile of Rna14, a conserved component of the cleavage and polyadenylation factor I complex (Casañal et al. 2017). To account for differences in transcription profiles observed between the slow mutant and the wild-type strain (Fig. 2D), we normalized Rna14 ChIP values to RNAPII occupancy by performing simultaneous measurements of Rna14 and Rpb1 recruitment from the same chromatin preparations. Consistent with the idea that the RNAPII slow mutant favors premature termination of *nc-tgp1* transcription, Rna14 was strongly enriched around the proximal PAS of *nc-tgp1* in the slow mutant compared with the wild-type (Fig. 3A, see regions 2–3), whereas reduced Rna14 levels were detected near the distal poly(A) site of *nc-tgp1* (Fig. 3A, see regions 5–6). We also monitored the recruitment pattern of Seb1, a RNAPII-associated factor that is important for PAS selection and transcription termination of coding and noncoding genes (Lemay et al. 2016; Wittmann et al. 2017). Similar to Rna14, we observed a redistribution of Seb1 from the distal to the proximal PAS of *nc-tgp1* (Fig. 3B). These results suggest that the slower elongation rate of the Rpb1 N494D mutant enhances the cotranscriptional recognition of suboptimal proximal PASs in *nc-tgp1*, leading to premature transcription termination.

RT-qPCR and ChIP assays were also used to assess whether APA-dependent premature termination of lncRNA transcription was responsible for derepression of the other core PHO regulon genes, namely, *pho84* and *pho1*. Importantly, RT-qPCR analysis confirmed the derepression of *pho84* and *pho1* in the *rpb1* mutant (amplicons 6 and 12–13, respectively) (Supplemental Fig. S6C,D), whereas the *prt2* and *nc-pho1* lncRNAs were both down-regulated (amplicons 3–4 and 8–11, respectively). Similar to the mechanism of *tgp1* derepression, RNAPII and Rna14 ChIP data support that the *prt2* lncRNA undergoes premature transcription termination in the *rpb1* slow mutant, leading to the activation of *pho84* (Supplemental Fig. S6E,F). However, it remains unclear whether such a mechanism also applies to the *nc-pho1/pho1* locus. Indeed, the proximity between the 3′ end of the *pho84* gene and the *nc-pho1* promoter makes the RNAPII and Rna14 ChIP data difficult to interpret (Supplemental Fig. S6E,F). One possibility is that the derepression of *pho84* as a consequence of premature termination of the *prt2* lncRNA might result in transcriptional interference at the *nc-pho1* promoter, thereby repressing *nc-pho1* transcription and ultimately coupling *pho84* and *pho1* derepression.

Next, we wondered which molecular feature of the Rpb1 N494D protein might lead to an increase in premature termination at the *nc-tgp1* locus. Since CTD Ser2 phosphorylation (Ser2P) coincides with the recruitment of the 3′ end processing machinery (Ahn et al. 2004; Meinhart and Cramer 2004), we analyzed CTD Ser2P levels across the *nc-tgp1* gene in wild-type and *rpb1-N494D* cells. ChIP experiments were performed using an antibody specific for CTD Ser2P (Larochelle et al. 2018) and normalized to levels of total Rpb1 in the respective regions. Although total levels of Ser2 CTD phosphorylation were similar between the wild-type and the slow mutant (Supplemental Fig. S7A), decreased levels of CTD Ser2P were detected at the distal PAS (regions 5–6) of *nc-tgp1* together with a concomitant increase at the proximal PAS (Fig. 3C, see region 2). As Ser2P is strongly associated with 3′ end processing, increased levels of this CTD modification near the proximal PAS of *nc-tgp1* is consistent with premature termination of *nc-tgp1* transcription in the RNAPII slow mutant. We also examined the levels of Thr4 and Ser7 CTD phosphorylation at the *nc-tgp1/tgp1* locus, as antagonistic functions in transcription termination were reported for these CTD modifications in fission yeast (Sanchez et al. 2018b). Specifically, Ser7P and Thr4P were shown to have negative and positive effects, respectively, on transcription termination. Consistent with these findings, the RNAPII slow mutant showed decreased levels of Ser7P concurrent with increased Thr4P signal at the *nc-tgp1* locus (Supplemental Fig. S7C,D) where we found increased levels of premature transcription termination in the slow mutant (Figs. 2, 3). The levels of Ser5 CTD phosphorylation across the *nc-tgp1* locus showed a slight redistribution toward the distal PAS in *rpb1-N494D* cells (Supplemental Fig. S7E), which could be a consequence of *tgp1* transcriptional activation.

To assess whether premature termination of *nc-tgp1* transcription was sufficient to induce *tgp1* expression, we designed a strategy to strengthen the proximal PAS of *nc-tgp1*. Upon examination of the sequences surrounding the two major *nc-tgp1* poly(A) signals, we noticed that whereas consensus 5′-GUA-3′ Seb1-binding motifs (Lemay et al. 2016) were found downstream from the distal PAS, there was no Seb1 motif downstream from the proximal PAS (Fig. 3D). Because Seb1 promotes RNAPII pausing, poly(A) site selection, and transcription termination (Lemay et al. 2016; Wittmann et al. 2017; Parsa et al. 2018), we reasoned that inserting a cluster of Seb1-binding sites downstream from the proximal PAS would increase the usage of the proximal site, thereby promoting premature termination of *nc-tgp1* transcription. We therefore used genome editing by CRISPR/Cas9 to insert five consensus Seb1-binding sites downstream from the proximal PAS of *nc-tgp1* (Fig. 3D, see *pPAS +5seb1*). Notably, the insertion of Seb1-binding motifs downstream from the proximal *nc-tgp1* PAS resulted in increased *tgp1* expression together with reduced levels of *nc-tgp1* lncRNA (Fig. 3E). Thus, by imposing RNAPII pausing downstream from the proximal *nc-tgp1* PAS, thereby promoting 3′ end processing and transcription termination, *tgp1* expression was induced even in phosphate-rich conditions.

### Recruitment of the Pho7 transcription factor at the tgp1 promoter is increased in the RNAPII slow mutant

**Figure 4.**
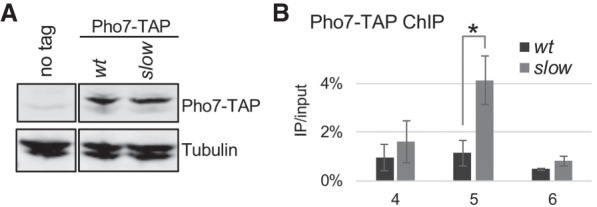
Increased recruitment of Pho7 at the *tgp1* promoter in the *rpb1* slow mutant. (*A*) Western blot analysis of TAP-tagged Pho7 in the wild-type and slow mutant with tubulin as a loading control. The image for the no-tag control strain was cropped from the same blot as the other two samples. (*B*) ChIP analysis of Pho7-TAP binding at the *tgp1* promoter (amplicons 4–6, as described in Figs. 2, 3) in the wild-type and slow mutant. The error bars represent standard deviation of the mean from three independent experiments. The difference between the slow and wild-type strains is significant at region 5 (two-sided Student's *t*-test *P-*value = 0.019).

It was previously noted that the distal PAS of *nc-tgp1* coincides with a binding site in the *tgp1* promoter for Pho7 (Sanchez et al. 2018a), a key transcription factor that controls phosphate homeostasis in fission yeast. Such overlap between the lncRNA transcription unit and the *tgp1* promoter argues for transcriptional interference (Ard et al. 2014; Ard and Allshire 2016). Accordingly, premature transcription termination of the *nc-tgp1* lncRNA gene in the RNAPII slow mutant (Figs. 2, 3) is expected to provide accessibility for Pho7 recruitment at the *tgp1* promoter. To examine Pho7 binding at the *tgp1* promoter in wild-type and *rpb1-N494D* cells, we analyzed chromatin occupancy of a TAP-tagged version of Pho7 (Fig. 4A) by ChIP assays. Despite equal expression of Pho7-TAP in both strains (Fig. 4A), there was a fourfold increase in Pho7 occupancy at the *tgp1* promoter in the RNAPII slow mutant relative to the wild-type control (Fig. 4B, see region 5). We therefore conclude that induction of the *tgp1* mRNA in the RNAPII slow mutant (Fig. 1) is the direct consequence of Pho7-dependent transcriptional activation.

### Premature termination of nc-tgp1 transcription triggers tgp1 induction upon phosphate starvation

**Figure 5.**
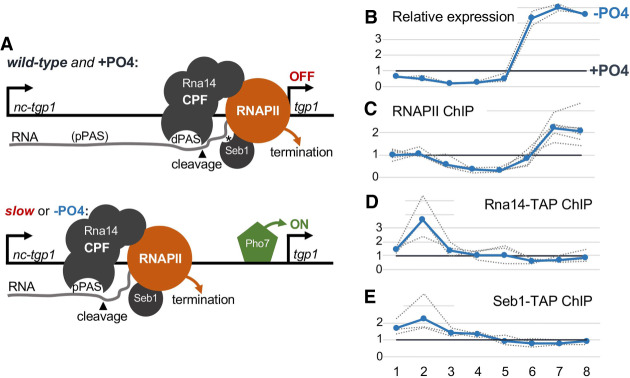
Phosphate-dependent regulation of *tgp1* expression is controlled by premature termination of *nc-tgp1* transcription. (*A*) Model for *tgp1* regulation by alternative cleavage and polyadenylation of its upstream noncoding RNA *nc-tgp1* (see text for details). (*) seb1-binding site; (CPF) cleavage and polyadenylation factor complex. (*B*–*E*) Wild-type cells grown in phosphate-containing minimal media (+PO4) were shifted into either +PO4 or phosphate-free (−PO4) minimal media during 4 h for all experiments. The results are expressed relative to the +PO4 condition after the 4-h incubation. Individual replicates are shown as dashed lines and their average is displayed as a thick blue line. The amplicons 1–8 target the *nc-tgp1/tgp1* locus, as described in Figures 2 and 3. (*B*) RT-qPCR analysis of *nc-tgp1/tgp1* expression upon phosphate depletion from two independent experiments. (*C*–*E*) Relative chromatin occupancy of Rpb1 (*C*), Rna14 (*D*), and Seb1 (*E*) at the *nc-tgp1/tgp1* locus upon phosphate depletion from six (*C*) or three (*D*,*E*) independent experiments. Individual replicates were normalized to total RNAPII levels, as measured from the same chromatin preparation.

The aforementioned results suggest a model whereby the slower elongation rate of the Rpb1 N494D mutant favors the cotranscriptional utilization of proximal PASs in *nc-tgp1*, thereby promoting premature termination of *nc-tgp1* transcription, which in turn allows for *tgp1* promoter activation by the Pho7 transcription factor (see Fig. 5A). To examine whether premature termination of *nc-tgp1* transcription is used to activate *tgp1* upon nutrient starvation, we analyzed *tgp1* expression in phosphate-rich (+PO_4_) and phosphate-deprived (−PO_4_) conditions using wild-type cells. As expected, the levels of *tgp1* mRNA increased after phosphate starvation (Fig. 5B, amplicons 6–8). In contrast, levels of the *nc-tgp1* lncRNA were substantially decreased in the absence of phosphate (Fig. 5B, amplicons 1–5). To assess whether the loss of the lncRNA upon phosphate starvation was due to premature transcription termination of *nc-tgp1*, we monitored RNAPII occupancy along the *nc-tgp1* and *tgp1* loci in +PO_4_ and −PO_4_ conditions. As shown in Figure 5C, Rpb1 levels declined downstream from the proximal PAS in phosphate-starved cells relative to cells grown in phosphate-rich conditions (regions 2–5), consistent with premature termination of *nc-tgp1* transcription induced by a phosphate deficiency. In agreement with this, the levels of Rna14 and Seb1 3′ end processing/transcription termination factors were both redistributed toward the proximal PAS of *nc-tgp1* in phosphate-deficient cells (Fig. 5D,E). Therefore, in conditions of phosphate limitation, transcription is prematurely terminated at the *nc-tgp1* locus to allow for induction of *tgp1* expression (see model in Fig. 5A).

### Widespread changes in alternative polyadenylation (APA) in the RNAPII slow mutant

**Figure 6.**
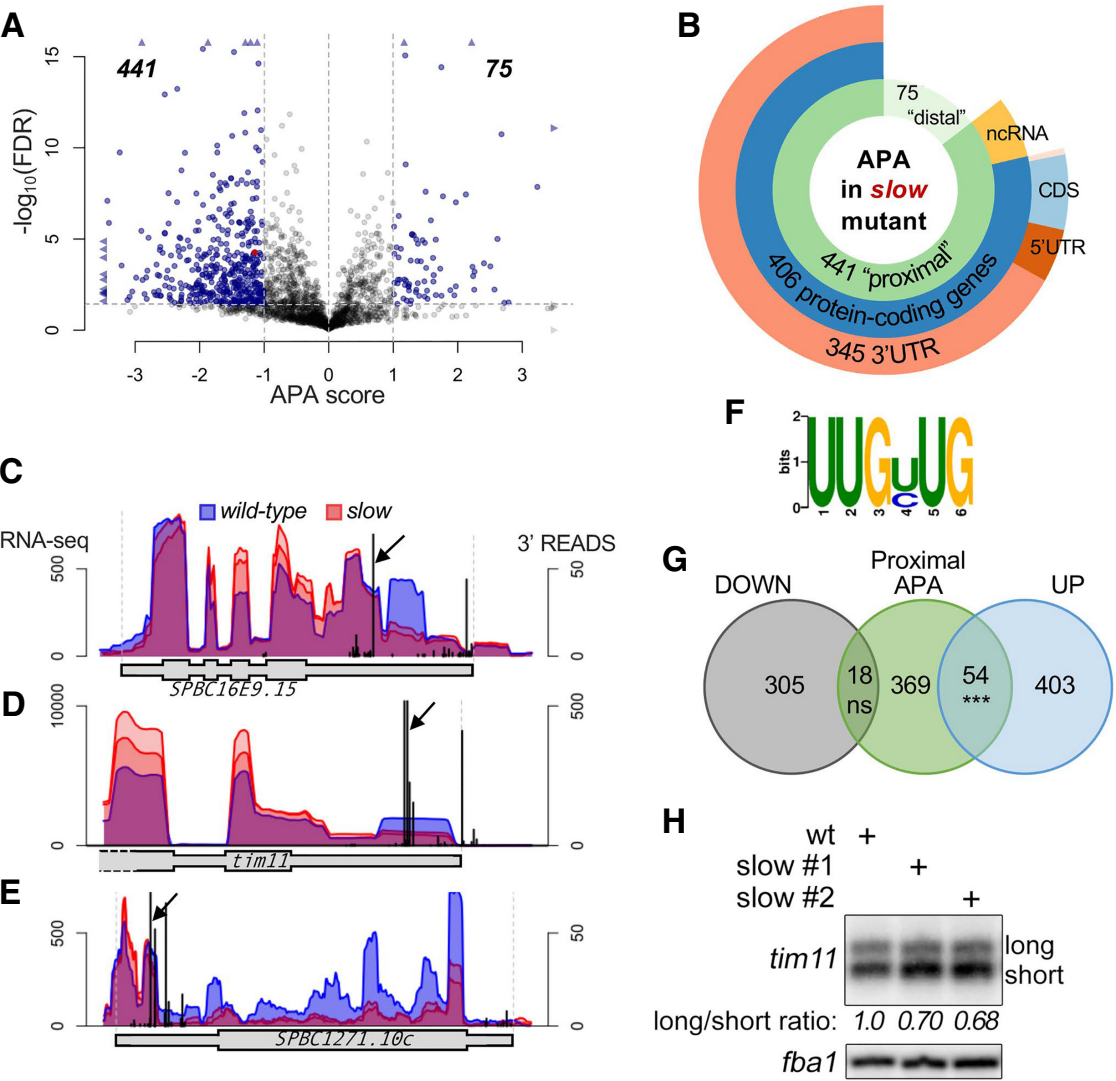
Widespread changes in poly(A) site selection in the *rpb1* slow mutant. (*A*) Volcano plot of statistical significance (−log_10_ of false discovery rate, FDR) against the APA score defined as the log_2_ ratio of distal to proximal segment ratio between the slow mutants and the wild-type (*n* = 2208 genes with at least two independent PASs) (see the Materials and Methods). Dashed lines represent the thresholds to select significant APA events (in blue); absolute APA score >1 and FDR <0.01. The single red dot represents *nc-tgp1*. To ease viewing, genes with values beyond axes limits are represented by arrowheads. (*B*) Distribution of the significant APA events identified among gene features. The terms “proximal” and “distal” APA describe genes for which the slow mutation favors proximal (negative APA score) and distal (positive APA score) PAS usage, respectively. (*C*–*E*) Normalized read coverage (*left* axis) at three different loci for the wild-type (blue area) and slow mutants (red areas) from RNA-seq data, and for previously published 3′ READS data (Liu et al. 2017b) in wild-type cells (black peaks; *right* axis). Black arrows indicate the proximal PAS at which the read coverage decreases in the slow mutant. Transcription is 5′ to 3′, *left* to *right*. (*F*) Sequence logo of UUG[UC]UG motif enriched downstream from affected versus unaffected proximal PAS. (*G*) Overlap between genes for which the RNAPII slow mutant significantly favors proximal (APA score <−1) poly(A) sites and the genes significantly down-regulated and up-regulated in the slow mutant. (***) *P*-value = 4.98×10^−06^; (ns) ot significant. (*H*) Northern blot analysis using a probe specific to *tim11* highlighting the long (634-nt) and short (539-nt) isoforms. The average ratio (*n* = 3) between both isoforms, normalized to the wild-type parental strain, is indicated in italic *below* each lane.

Most genes in eukaryotes contain multiple cleavage/polyadenylation sites, which can be dynamically regulated during cell proliferation and differentiation as well as in response to various stresses (Mayr 2016; Tian and Manley 2017). Our data support that APA coupled to transcription termination of an upstream lncRNA gene is the mechanism responsible for *tgp1* activation in the RNAPII slow mutant as well as in conditions of phosphate starvation in fission yeast. To measure the global effect of a slow transcription elongation rate on APA, we examined the changes in poly(A) site selection from our transcriptomic data of the slow mutant and control strain for genes with at least two independent PASs (*n* = 2208). Specifically, we calculated a distal/proximal ratio from the RNA-seq read coverage for genomic regions based on proximal and distal PAS usage (Liu et al. 2017b) and compared this ratio between the wild-type and the RNAPII slow mutant to get an APA score. In total, we found 516 genes that showed a significant change in APA score in the slow mutant relative to wild-type cells (Fig. 6A; Supplemental Table S2). Notably, of the 516 APA-sensitive genes identified in the RNAPII slow mutant, 441 (85%) showed a shift toward increased usage of the proximal PAS, including the *nc-tgp1* lncRNA (Fig. 6A, red dot). Most of the 441 APA events that showed a distal-to-proximal shift in the slow mutant took place in the 3′ UTR of protein-coding genes (Fig. 6B), as shown for *SBPC16E9.15* (Fig. 6C) and *tim11* (Fig. 6D). We also found cases in which the RNAPII slow mutant showed reduced read coverage downstream from poly(A) sites clustered in the 5′ UTR of genes (Fig. 6E), suggesting that premature transcription termination close to transcription start sites is used to control gene expression in fission yeast.

Altogether, we found that ∼20% (441 out of 2208) of genes with at least two independent PASs showed a shift toward the preferential use of the proximal PAS in the *rpb1* slow mutant (Fig. 6A,B). To examine the possibility that differences in the sequences surrounding the affected PAS could explain why a subset of genes are particularly sensitive to a reduction in transcription elongation rate, DREME (Bailey 2011) was used to compare motif sequences 40 nt upstream of and downstream from the proximal cleavage sites of affected and unaffected genes. No significant differences were observed upstream of the cleavage sites, but a U-rich motif (Fig. 6F) was found to be differentially enriched (*e*-value = 3.5 × 10^−2^) downstream from the cleavage sites of affected genes. Analysis of nucleotide frequencies also revealed that the regions immediately upstream of and downstream from the A-rich cleavage sites of proximal PASs of genes affected by the slow mutant were significantly enriched in uridine (*P* = 3.084 × 10^−8^) compared with proximal PAS of unaffected genes (Supplemental Fig. S8). These observations support the notion that specific genes tend to share certain sequence motifs surrounding the proximal PAS, which may allow them to be particularly sensitive to a reduction in transcription elongation rate.

We next examined the connection between APA-sensitive events and changes in gene expression. We observed a significant overlap between genes that showed distal-to-proximal APA and those that were up-regulated in *rpb1-N494D* cells (Fig. 6G). Within this overlapping module, computational analyses (Bitton et al. 2015) to examine for enrichment of functional gene classes revealed that genes involved in the purine nucleotide biosynthesis process were significantly over-represented (*P*-value 5 × 10^−06^; odds ratio 2.15). An example of this is shown for *tim11*, a gene encoding a subunit of the F1-FO ATP synthase, for which we noted decreased read coverage downstream from the proximal poly(A) site together with a twofold increase in read coverage inside the gene body in the slow mutant (Fig. 6D). The effect of the RNAPII slow mutant on *tim11* poly(A) site selection was confirmed by Northern blotting (Fig. 6H). Our data thus indicate that the slow transcription elongation rate in *rpb1-N494D* cells causes widespread distal-to-proximal shifts in APA, affecting the expression of a specific set of genes, including the core PHO regulon (indirectly via upstream lncRNA) and genes involved in the purine biosynthesis pathway (via 3′ UTR shortening).

### Limiting nucleoside triphosphate concentration activates phosphate-responsive genes

**Figure 7.**
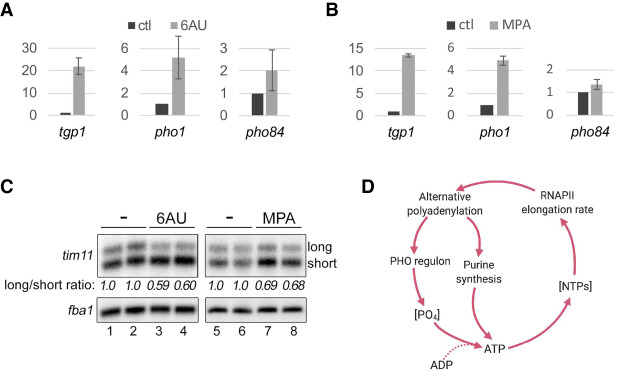
Limiting NTP concentration activates phosphate-responsive genes. (*A*,*B*) RT-qPCR analysis of *tgp1*, *pho1*, and *pho84* expression relative to *nda2* in response to 6AU and MPA. Cells were grown in EMM and treated for 1 h with either 30 µg/mL of 6-azauracil (6AU) (*A*) or 30 µg/mL of mycophenolic acid (MPA) (*B*) or left untreated (ctl). Error bars represent the standard deviation of the mean over two independent experiments. (*C*) Northern blot analysis of *tim11* short and long isoforms. Total RNA prepared from wild-type cells that were treated with 6AU (lanes *3*,*4*) or MPA (lanes *7*,*8*), as described in *A* and *B*, was analyzed by Northern blotting. Control cells were treated with DMSO (lanes *1*,*2*) or methanol (lanes *5*,*6*). Independent replicates are shown (cf. lanes *1* and *3*, *2* and *4*, *5* and *7*, and *6* and *8*). The average ratio between both isoforms, normalized to the ratio in untreated control cells, is indicated in italic *below* each lane. (*D*) Model of homeostatic regulation of phosphate-responsive genes by control of RNAPII elongation kinetics and APA (see the text for details).

Our results reveal that fission yeast respond to conditions of both phosphate starvation and slow transcription elongation by inducing the expression of a core PHO regulon via a mechanism that involves APA and premature transcription termination of interfering lncRNAs. To elucidate the molecular events that elicit this gene activation response, we considered that a deficiency in inorganic phosphate results in depletion of the intracellular NTP pool (Boer et al. 2010; Ljungdahl and Daignan-Fornier 2012). Notably, when NTP substrate concentrations are reduced by treating cells with drugs such as 6AU and MPA that impair nucleotide biosynthesis (Exinger and Lacroute 1992; Shaw and Reines 2000), the elongation rate of RNAPII decreases (Mason and Struhl 2005). Thus, we reasoned that *tgp1, pho1,* and *pho84* genes may have evolved to be positively regulated by a reduced rate of transcription elongation, which is anticipated in conditions of phosphate starvation as a consequence of limiting NTP concentration. To test whether limiting NTP concentration results in the activation of the core PHO regulon, we treated wild-type cells with 6AU and MPA. As shown in Figure 7, A and B, 6AU and MPA both resulted in increased levels of the core PHO regulon genes, although the effect of MPA on *pho84* expression was minimal. NTP depletion using 6AU or MPA also resulted in increased levels of the short 3′ UTR version of *tim11* relative to the long 3′ UTR isoform (Fig. 7C), consistent with increased usage of proximal poly(A) sites as a consequence of reduced transcription elongation rates. Our results thus support the notion that decreased intracellular NTP concentrations as a consequence of nutrient limitations can result in an overall reduction in the transcription elongation rate, which in turn can activate genes that are particularly sensitive to slower RNAPII kinetics, such as genes that promote immediate phosphate uptake (core PHO regulon) and purine biosynthesis.

## Discussion

It is now widely appreciated that the rate of RNAPII elongation is highly dynamic, varying extensively both within and between genes (Danko et al. 2013; Jonkers et al. 2014; Veloso et al. 2014; Cortazar et al. 2019). To date, however, how the transcription elongation rate is controlled still remains poorly understood. Furthermore, it remains unknown whether changes in the elongation rate of RNAPII are used to control and coordinate gene expression. In this study, we used a previously characterized *rpb1* mutant (Malagon et al. 2006; Jimeno-González et al. 2010; Strathern et al. 2013) to investigate how a slow transcription rate globally affects gene expression. Intuitively, a reduced rate of transcription elongation is expected to result in a general decrease in mRNA synthesis. However, although our RNA-seq analyses revealed a set of genes with significantly reduced RNA accumulation, the large majority of genes showed normal steady-state RNA levels in the RNAPII slow mutant (Fig. 1). This can be explained, at least in part, by a general decrease in RNA decay rates, which apparently compensates for the decreased rate of mRNA synthesis in this *rpb1* slow mutant (Sun et al. 2012). On the other hand, the up-regulation of more than 450 transcripts (Fig. 1), including a core set of genes involved in phosphate acquisition and purine synthesis, in a mutant with a reduced rate of transcription elongation was mostly unexpected. By studying how the expression of the phosphate-responsive permease gene *tgp1* is up-regulated in the *rpb1* slow mutant, we unveiled a mechanism that involves alternative polyadenylation (APA) and premature transcription termination of an upstream interfering lncRNA (Fig. 5A) and showed that this mechanism is occurring naturally to activate *tgp1* expression in conditions of phosphate starvation. Importantly, our findings argue that variations in the transcription elongation rate is a critical mode by which cells control their transcriptome in response to nutrient limitations (Fig. 7D).

### Transcription elongation kinetics regulate alternative polyadenylation (APA)

Most eukaryotic genes harbor multiple poly(A) sites and a growing number of mechanisms have been shown to control APA, including core 3′ end processing factors and specific RNA-binding proteins (Mayr 2017; Tian and Manley 2017). Previous findings in *Drosophila* suggest that kinetic competition between RNAPII elongation and 3′ end processing influences poly(A) site selection (Pinto et al. 2011; Liu et al. 2017a). Mechanistically, RNAPII speed would regulate APA by restraining the competition between alternative PASs: A slow elongation rate would favor proximal PAS usage by increasing the window of time that this PAS is available for cleavage by the 3′ end processing machinery before a competing distal PAS is transcribed. Our RNA-seq analysis of the *rpb1-N494D* mutant is totally consistent with this view, revealing that a reduction in the transcription elongation rate enhances proximal PAS usage (Fig. 6). It therefore appears that RNAPII elongation kinetic is a critical parameter that influences the efficiency of most cotranscriptional RNA processing events, including splicing (Roberts et al. 1998; de la Mata et al. 2003; Fong et al. 2014; Aslanzadeh et al. 2018), poly(A) site selection (this study; Pinto et al. 2011; Liu et al. 2017a), and transcription termination (Hazelbaker et al. 2013; Fong et al. 2015; Cortazar et al. 2019).

### Gene activation by APA-dependent premature termination of lncRNA transcription

In yeast, a conserved set of phosphate-responsive genes (core PHO regulon) provides the initial response to phosphate starvation by rapidly inducing *pho1*, *pho84*, and *tgp1* (Carter-O'Connell et al. 2012), whose protein products are responsible for immediately harvesting inorganic phosphate from the environment. Interestingly, these three genes have been shown to be repressed by upstream lncRNA-mediated transcription in fission yeast (Ard et al. 2014; Shah et al. 2014; Garg et al. 2018; Sanchez et al. 2018a). In recent years, there has been an increasing number of examples of negative gene regulation by adjacent transcription of a lncRNA, a process often referred to as transcriptional interference, which has been described in yeast, fruit flies, mice, and humans (Ard et al. 2017). Transcriptional interference generally depends on refractory changes caused by the transcription of an upstream noncoding RNA in the chromatin architecture of a downstream protein-coding gene promoter. Although numerous mechanisms whereby the act of lncRNA transcription represses a downstream gene have been described (Ard et al. 2017; Kaikkonen and Adelman 2018), how these systems are reversibly inactivated to allow induction of the protein-coding gene has remained poorly understood. In this study, we found that the core PHO regulon in *S. pombe* is activated by a mutant RNAPII with reduced elongation rate. Several lines of evidence support that activation of *tgp1*, a core PHO regulon gene in *S. pombe*, is mediated by premature termination of upstream lncRNA transcription. First, reduction of transcription elongation rate in the *rpb1-N494D* mutant caused a decline in RNAPII occupancy beyond the proximal *nc-tgp1* PAS, which coincided with reduced production of *nc-tgp1* lncRNA (Fig. 2), increased Pho7 binding at the *tgp1* promoter (Fig. 4), and induction of *tgp1* mRNA (Figs. 1,2). Second, slower RNAPII elongation resulted in the redistribution of 3′ end processing/termination factors from the distal to the proximal PAS of the *nc-tgp1* lncRNA (Fig. 3). Third, enforcing RNAPII pausing and transcription termination at the proximal *nc-tgp1* PAS was sufficient to induce *tgp1* expression in phosphate-rich conditions (Fig. 3D,E). Finally, a mutation altering the proximal poly(A) signal of *nc-tgp1* was shown to attenuate *tgp1* induction (Sanchez et al. 2018a).

Collectively, these findings support a model of gene regulation by lncRNA-mediated transcriptional interference that is controlled by APA and premature termination of lncRNA transcription (Fig. 5A). In wild-type cells, an optimal rate of transcription elongation together with the absence of Seb1-binding motifs downstream from the proximal PAS results in suboptimal utilization of the proximal PAS relative to the distal PAS during transcription of *nc-tgp1* (Fig. 5A, top panel). As the distal *nc-tgp1* PAS directly overlaps with the binding site for the transcription factor Pho7 (Schwer et al. 2017), transcription and 3′ end processing at the distal *nc-tgp1* PAS is likely to impinge on the binding of Pho7 to the *tgp1* promoter. Transcription-coupled chromatin modifications may also contribute to repress *tgp1* expression via lncRNA transcription through its promoter, a mechanism shown previously for *tgp1* (Ard and Allshire 2016) and other fission yeast genes (Shah et al. 2014; Touat-Todeschini et al. 2017). In the context of a slower transcription elongation rate, such as in *rpb1-N494D* cells, the frequency of proximal PAS usage is increased relative to the distal PAS, leading to premature termination of *nc-tgp1* transcription, which prevents lncRNA transcription through the downstream *tgp1* promoter, thereby allowing for the recruitment of the Pho7 transcription factor and *tgp1* induction (Fig. 5A, bottom panel).

Importantly, we also show that phosphate-dependent regulation of *tgp1* expression is controlled by APA and premature termination of the upstream *nc-tgp1* lncRNA (Fig. 5). This is in contrast to previous examples of lncRNA-mediated gene regulation where the expression of the lncRNA is regulated at the level of transcriptional initiation (Martens et al. 2004, 2005; Hirota et al. 2008). Indeed, our ChIP data indicate similar levels of RNAPII at the *nc-tgp1* promoter between the wild-type and the slow mutant (Fig. 2D, see region 1) as well as between phosphate-rich and phosphate-deficient conditions (Fig. 5C, region 1). Thus, although we cannot completely rule out some level of transcriptional control of the *nc-tgp1* lncRNA promoter in response to phosphate concentration, our results argue that APA-dependent premature termination of noncoding RNA transcription is a crucial mechanism of gene activation for the core PHO regulon in *S. pombe*.

While premature 3′ end processing is a widespread phenomenon in the RNAPII slow mutant (Fig. 6), our RNA-seq data indicated that a small number of ncRNAs (*n* = 35) relative to protein-coding genes (*n* = 406) showed premature 3′ end processing. Although *nc-tgp1/tgp1* and *prt2/pho84* provide strong cases where premature termination of an upstream interfering ncRNA affects the expression of a downstream gene, manual inspection of the remaining ncRNAs affected in the RNAPII slow mutant did not provide clear evidence for activation of a neighboring protein-coding gene. However, it remains possible that regulation of transcription elongation rate at ncRNA genes may modulate the expression of adjacent protein-coding genes by other mechanisms than premature termination. For instance, the RNA-seq analysis of the RNAPII slow mutant revealed the induction of *ste11*, which encodes a master transcription factor for sexual development that activates a number of genes required for mating and meiosis in fission yeast. Interestingly, similar to the core PHO regulon genes, *ste11* is induced by nutrient starvation and is negatively regulated by a lncRNA. In the case of *ste11*, gene repression is mediated in *cis* by a divergently transcribed lncRNA, *rse1*, that recruits a protein complex that promotes deacetylation of the *ste11* promoter (Fauquenoy et al. 2018). As a secondary structure in the divergently transcribed *rse1* lncRNA appears to tether the repressive complex that deacetylates the *ste11* promoter (Fauquenoy et al. 2018), it is tempting to speculate that RNAPII elongation rate could influence the efficiency of cotranscriptional lncRNA folding, which can have crucial consequences on the recruitment of RNA-binding proteins. Consistent with this view, the speed of transcription elongation has been shown to affect riboswitch folding in bacteria (Wickiser et al. 2005; Chauvier et al. 2017) and formation of a stem–loop structure important for histone pre-mRNA processing in humans (Saldi et al. 2018).

### Transcription elongation rate controls a gene expression program in response to phosphate starvation

An unexpected finding from our study was that the preferential use of proximal PASs in the RNAPII slow mutant resulted in increased expression of genes involved in two important and related cellular pathways: phosphate acquisition and purine biosynthesis. What is the physiological relevance of decreased transcription speed increasing the expression of the core PHO regulon and genes involved in purine biosynthesis? Interestingly, purine and phosphate pathways are known to be coregulated in budding and fission yeasts (Gauthier et al. 2008; Estill et al. 2015), but the underlying mechanism linking the two pathways has remained unclear. A key role of phosphate assimilation in cells is the phosphorylation of ADP to ATP, a purine required for nucleic acid synthesis and a central energy supply (Ljungdahl and Daignan-Fornier 2012). Accordingly, phosphate starvation in yeast causes a drastic deficiency in ATP, but also in UTP and CTP (Boer et al. 2010). Since these NTPs are direct substrates of RNAPII during RNA synthesis, limiting intracellular NTP concentrations causes a decrease in the rate and processivity of RNAPII (Mason and Struhl 2005). Our data therefore support a model (Fig. 7D) whereby nutrient availability modulates the elongation rate of RNAPII to control the activation of specific gene expression programs. In the case of phosphate starvation, we propose that limitation for extracellular phosphate results in intracellular NTP depletion, causing a global reduction in transcription elongation rate. Importantly, we have shown that a reduction in RNAPII elongation rate enhances proximal PAS usage by APA, resulting in activation of genes specifically needed in response to phosphate starvation: the core PHO regulon and genes involved in purine biosynthesis (Fig. 7D). Interestingly, links between the nutritional status of the cell, NTP concentration, and transcription have been previously established in budding yeast. Accordingly, NTP sensing by the transcription machinery provides a mechanism to control the expression of genes involved in specific nucleotide biosynthesis pathways; however, in this case, limiting NTP concentration control RNAPII initiation at specific promoters (Davis and Ares 2006; Thiebaut et al. 2008; Kuehner and Brow 2019). The identification of a mechanism of gene activation that responds to nutrient limitation by altering RNAPII elongation kinetics, as reported here, thus constitutes a new regulatory paradigm that could allow cells to meet the rapidly changing needs in nutrient availability. Quite remarkably, our results therefore suggest that subsets of genes have evolved specific mechanisms that allow them to be particularly responsive to a slow transcription elongation rate. For the core PHO regulon genes, we provide a molecular explanation for how APA-mediated premature termination of upstream lncRNA transcription allow for their activation in cells with a reduced rate of RNAPII elongation. In the case of genes involved in purine biosynthesis, future work will determine how 3′ UTR shortening of encoded mRNAs can lead to increased expression upon reduced RNAPII elongation kinetics.

In summary, our findings support the notion that the nutritional state of eukaryotic cells, reflected by NTP concentrations, can alter the rate and processivity of RNAPII elongation, which in turn influence the efficiency of RNA processing events such as poly(A) site selection to regulate specific expression programs. Given the primordial role of alternative splicing and alternative polyadenylation in mammalian cells, it will be interesting to see whether variations in the RNAPII elongation rate in responses to changes in the nutritional status or the during cellular differentiation can also activate specific expression programs in higher eukaryotes.

## Materials and methods

### Yeast strains and media

A list of all *S. pombe* strains used in this study is provided in Supplemental Table S3. Unless stated otherwise, cells were grown to mid-log phase (OD_600_ 0.5–0.7) at 30°C in YES or EMM medium supplemented with adenine, histidine, leucine, and uracil. CRISPR/Cas9-mediated mutagenesis was used to generate the *rpb1-N494D* and +*5seb1* mutants by applying the “fluoride-selection” (Fernandez and Berro 2016) or the “short homology” protocol (Hayashi and Tanaka 2019), respectively. C-terminal tagging of proteins were performed by PCR-mediated gene targeting (Bähler et al. 1998) using lithium acetate method for yeast transformation.

### Growth assays

Mycophenolic acid (MPA; Sigma m3536) and 6-azauracil (6AU; Sigma A1757) were resuspended in methanol and DMSO, respectively, to a stock concentration of 50 mg/mL. As the large quantity of uracil contained in uracil-supplemented media neutralizes the effect of 6AU (Reines 2003), the tested *S. pombe* strains were made autotroph for uracil by transformation with *ura4*-containing plasmids (FB168). Exponentially growing cultures of those ura4+ strains were concentrated to an OD_600_:1 and serially diluted fivefold in the appropriate liquid media. Each dilution was spotted on freshly prepared plates containing the indicated concentration of drug or an equal volume of solvent.

### Western blots

Total cell extracts were prepared by harvesting cells in mid-log phase in ice-cold lysis buffer (50 mM Tris at pH 7.5, 5 mM MgCl2, 150 mM NaCl and 0.1% NP-40) containing a cocktail of protease inhibitors (1× PMSF, 1× PLAAC) with half of a phosphor STOP tablet, prior to lysis with glass beads using a FastPrep instrument (MP Biomedicals). Clarified lysates were normalized for total protein concentration using the Bradford protein assay. Thirty micrograms of total proteins was separated by SDS-PAGE, transferred to nitrocellulose membranes, and analyzed by immunoblotting using a rabbit polyclonal antibody against protein A (1:10,000 [v/v] dilution; Sigma-Aldrich, P3775). Membranes were then probed with goat anti-rabbit secondary antibodies conjugated to IRDye 800CW (1:15,000 [v/v] dilution; LI-COR, 926-32213). Detection of the proteins was performed using an Odyssey infrared imaging system (LI-COR).

### Chromatin immunoprecipitation (ChIP) assays

For each IP, 50 mL of cells grown in EMM were incubated for 20 min at room temperature with 1% formaldehyde. After quenching the reaction with glycine (final concentration of 360 mM) for 5 min at room temperature, cells were washed twice with cold Tris-buffered saline (20 mM Tris–HCl at pH 7.5, 150 mM NaCl). Cell pellets from 50-mL cultures were resuspended in 500 μL of lysis buffer (50 mM HEPES-KOH at pH 7.5, 140 mM NaCl, 1 mM EDTA at pH 8.0, 1% Triton X-100, 0.1% Na-deoxycholate) containing protease inhibitors and disrupted using a FastPrep instrument. Samples were then sonicated 10 times for 10 sec at 20% intensity using a Branson digital sonifier. Whole-cell extract (WCE; 500 μL) was incubated overnight at 4°C with 50 µL of magnetic beads only (PAN mouse IgG; Invitrogen) in the case of the immunoprecipitation of HTP/TAP-tagged proteins, or of magnetic beads coupled with 2 µg of one of the following antibodies: 8WG16 (unphosphorylated CTD), clone 3E10 (phospho CTD Ser2; Millipore), clone 3E8 (phospho CTD Ser5; Millipore), clone 3D4A12 (phospho CTD Ser7; Active Motifs), and clone 6D7 (phospho CTD Thr4; Active Motifs). Beads were washed twice with 1 mL of lysis buffer, twice with 1 mL of lysis buffer plus 500 mM NaCl, twice with 1 mL of wash buffer (10 mM Tris-HCl at pH 8.0, 250 mM LiCl, 0.5% NP-40, 0.5% sodium deoxycholate, and 1 mM EDTA), and once with 1 mL of Tris-EDTA (TE; 10 mM Tris-HCl at pH 8.0, 1 mM EDTA). Bound material was eluted by resuspending beads in 50 μL of elution buffer (50 mM Tris-HCl at pH 8.0, 10 mM EDTA, 1% SDS) after 15-min incubation at 65°C. After overnight incubation at 65°C for reverse-crosslinking, the samples were treated with proteinase K and cleaned up by phenol-chloroform extraction before undergoing a RNase treatment and a final purification on PCR purification column (Qiagen). DNA from the inputs and IPs were analyzed on a LightCycler 96 Instrument using perfecta SYBR supermix in the presence of a 150 nM concentration (each) of gene-specific primers in 15-μL reaction mixtures. Protein occupancy was then calculated using the percent input method (Lemay et al. 2014; Larochelle et al. 2018). For the IPs of phosphorylated CTD or the RNAPII-associated 3′ end processing/termination factors (Rna14 and Seb1), the values were further normalized to RNAPII occupancy measured from the same chromatin preparation, as previously described (Lemay et al. 2016; Larochelle et al. 2018).

### RNA preparation and analyses

Total RNA was extracted using the hot-acid phenol method (Lemay et al. 2014; Larochelle et al. 2018). For Northern blots, RNA samples were resolved on 2% agarose gels, transferred onto nylon membranes, and probed using ^32^P-labeled riboprobes. For RT-qPCR analyses, RNA samples (1 µg of total RNA) were treated with DNase RQ1 (Promega, M6101) and reverse transcribed using the Omniscript RT (Quiagen). Gene expression relative to the appropriate control strain was measured on a LightCycler 96 system (Roche) with the ΔΔ*CT* method using the gene *nda2* as internal reference (Lemay et al. 2014; Larochelle et al. 2018). RNase protection assays were performed as previously described (Mercier et al. 2008). T7 promoter-containing PCR products were used to produce antisense RNA probes that served to determine nc-tgp1 and *act1* RNA levels. ^32^P-labeled antisense RNA probes were produced using the aforementioned PCR products with the use of [α-^32^P]UTP and T7 RNA polymerase. The *act1* transcript was used as an internal control for normalization during quantification of the RNase protection products.

### Library preparation and Illumina sequencing

Library preparation was performed at the RNomics Platform of Université de Sherbrooke. Briefly, 10 µg of total RNA extracted from cells grown in EMM was treated with DNase on a RNeasy minikit column (Qiagen) according to the manufacturer's protocol. RNA integrity was assessed on a 2100 Bioanalyzer machine (Agilent Technologies). Ribo-depleted RNA was isolated from 1.25 µg of total RNA using Illumina RiboZero (yeast), as per manufacturer's protocol. Stranded RNA-seq libraries were then built using the SSV21106 kit (Epicentre) from the ribo-depleted RNA with 13 cycles of PCR amplification. After assessment of libraries quality and molarity on a DNA high-sensitivity chip (Agilent), the libraries were pooled and sequenced in paired-end (2 × 100 bp) on a HiSeq 4000 (Illumina) at the McGill University and Génome Québec Innovation Centre (MUGQIC) Sequencing Service with at least 250× genome depth (>35 millions reads) for each sample.

### RNA-seq data analysis

The reads were trimmed and aligned according to the MUGQIC RNA-seq pipeline (bitbucket.org/mugqic/genpipes/src/master/pipelines/rnaseq). Briefly, low-quality reads and adaptor sequences were removed with Trimmomatic v0.36 with parameters ILLUMINACLIP:adapters-truseq.fa:2:30:15:8:true TRAILING:30 MINLEN:32 (Bolger et al. 2014). The remaining reads were then aligned to the *S. pombe* genome assembly ASM294v2 with STAR v2.5.1 (Dobin et al. 2013) and summarized at the gene level using featureCounts with options -p -s 2 -P -d 0 -D 1000 -C -T 4 (Liao et al. 2014). Differential gene expression analysis was performed using the DESeq2 R package (v1.24) (Love et al. 2014). The AnGeLi tool (Bitton et al. 2015) was used to assess enrichment in biological processes of the differentially expressed genes identified. Raw and processed data are available on GEO under the accession number GSE140698.

### Alternative polyadenylation (APA) analysis

To estimate the change in poly(A) site selection from transcriptomic data, we first segmented genes in smaller windows delimited by peaks of 3′READS read counts (Liu et al. 2017b), representing PAS usage for a WT strain grown in EMM. The processed 3′READS data used in this study are accessible under accession number GSM2496939. To be considered in the segmentation process, 3′READS peaks had to be constituted of more than five reads and account for at least 5% of a given gene 3′READS total read coverage. Distinct peaks closer than 50 nt were merged together at the stronger peak position. For genes delimited by at least two segments, we divided the normalized (DESeq2 median ratios method) RNA-seq read coverage of the most distal segment (relative to transcription start site) by the normalized read coverage of the most proximal segment. Then, we compared this ratio between and the RNAPII slow mutants and the wild-type strain to obtain an APA score defined as *log_2_[(slow/wt)_distal_/slow/wt)_proximal_]*. Statistical significance of the difference in ratios between the slow mutants and the wild-type strain was assessed with a generalized linear model of the binomial family using the glm() function in R. This method allows one to take into account not only the change in PAS usage ratio, but also the normalized read counts of the numerator and denominator of the ratio, therefore accounting for the magnitude of count values in the calculation of statistical significance.

### Motif and sequence analysis

Affected proximal PAS (*n* = 441) were defined as having an APA score <1 and an associated adjusted *P*-value (FDR) <0.01. Proximal PAS whose APA score is close to 0, within thresholds (between −0.2215 and 0.02215) allowing the same number of PAS to be selected (441), served as the unaffected control group. Differential motif analysis was performed using DREME (Bailey 2011) and average base composition was summarized using FastQC.

## Supplementary Material

Supplemental Material
