# Supplementary material for: Nutrient-dependent control of RNA polymerase II elongation rate regulates specific gene expression programs by alternative polyadenylation
Source: Genes Dev. 2020 Jul 1;34(13-14):883–97. doi: 10.1101/gad.337212.120 (PMC7328516; doi:10.1101/gad.337212.120)
Supplement: Supplemental Material [file supp_34_13-14_883__index.html]

Nutrient-dependent control of RNA polymerase II elongation rate regulates specific gene expression programs by alternative polyadenylation — Supplemental Material 

# Nutrient-dependent control of RNA polymerase II elongation rate regulates specific gene expression programs by alternative polyadenylation

## Supplemental Material

- Supplemental\_FigS1.pdf
- Supplemental\_FigS2.pdf
- Supplemental\_FigS3.pdf
- Supplemental\_FigS4.pdf
- Supplemental\_FigS5.pdf
- Supplemental\_FigS6.pdf
- Supplemental\_FigS7.pdf
- Supplemental\_FigS8.pdf
- Supplemental\_TableS1.xlsx
- Supplemental\_TableS2.xlsx
- Supplemental\_TableS3.xlsx
