# Supplementary material for: Nutrient-dependent control of RNA polymerase II elongation rate regulates specific gene expression programs by alternative polyadenylation
Source: Genes Dev. 2020 Jul 1;34(13-14):883–97. doi: 10.1101/gad.337212.120 (PMC7328516; doi:10.1101/gad.337212.120)
Supplement: Supplemental Material [file supp_gad.337212.120_Supplemental_FigS1.pdf]

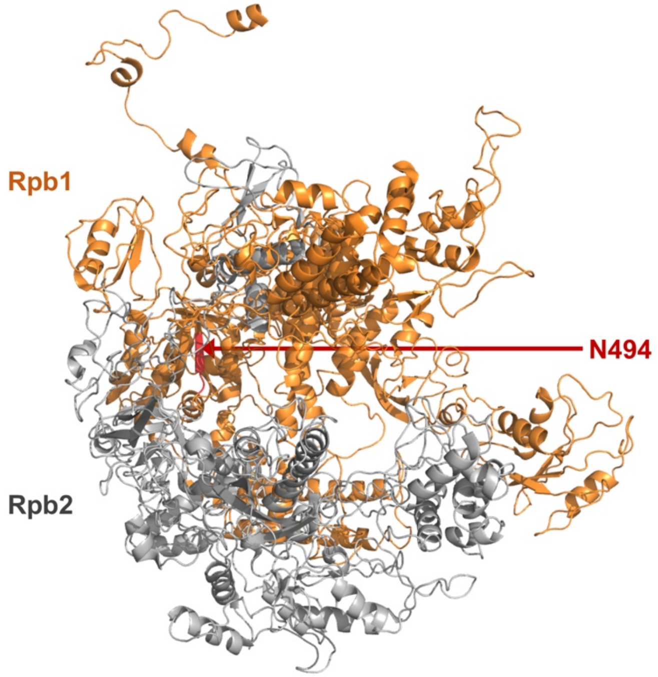

Supplemental Figure S1. **Structure of *S. pombe* RNAPII from X-ray diffractions at 3.65 Å resolution (*pdb3h0g,* (Spahr et al. 2009)) visualized using PyMOL**.

Only the two largest subunits, Rpb1 and Rpb2 in orange and grey, respectively, are shown. The position of the substituted residue in the slow mutant (Rpb1 N494) in the catalytic core of the polymerase is indicated by a red arrow.
