# Supplementary material for: Nutrient-dependent control of RNA polymerase II elongation rate regulates specific gene expression programs by alternative polyadenylation
Source: Genes Dev. 2020 Jul 1;34(13-14):883–97. doi: 10.1101/gad.337212.120 (PMC7328516; doi:10.1101/gad.337212.120)
Supplement: Supplemental Material [file supp_gad.337212.120_Supplemental_FigS2.pdf]

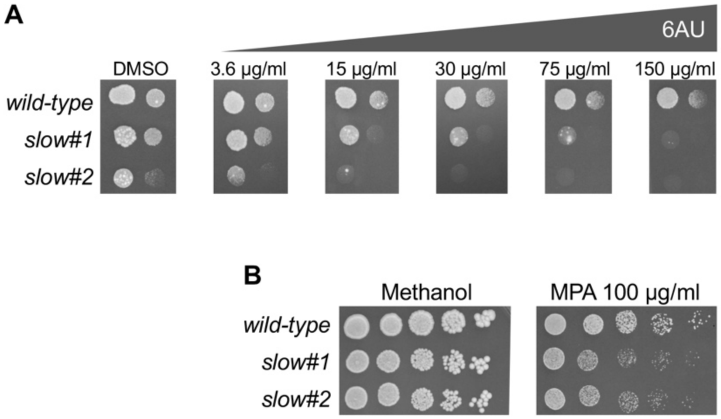

Supplemental Figure S2**. *rpb1-N494D* cells are hypersensitive to 6AU and MPA.**

Growth assays showing five-fold dilutions of the indicated strains grown on solid media for 5 days at 30°C. **(A)** Precultures of the indicated strains were grown in EMM -uracil media until mid-log phase and concentrated at OD_{600} = 1 before plating on EMM -ura plates supplemented with 6-azauracil (6AU) at the indicated concentrations or with equal volume of solvent (DMSO). **(B)** Precultures for the indicated strains were grown in YES media until mid-log phase and concentrated at OD_{600} = 1 before plating on YES plates supplemented with mycophenolic acid (MPA) or with equal volume of solvent (methanol).
