# Supplementary material for: Nutrient-dependent control of RNA polymerase II elongation rate regulates specific gene expression programs by alternative polyadenylation
Source: Genes Dev. 2020 Jul 1;34(13-14):883–97. doi: 10.1101/gad.337212.120 (PMC7328516; doi:10.1101/gad.337212.120)
Supplement: Supplemental Material [file supp_gad.337212.120_Supplemental_FigS3.pdf]

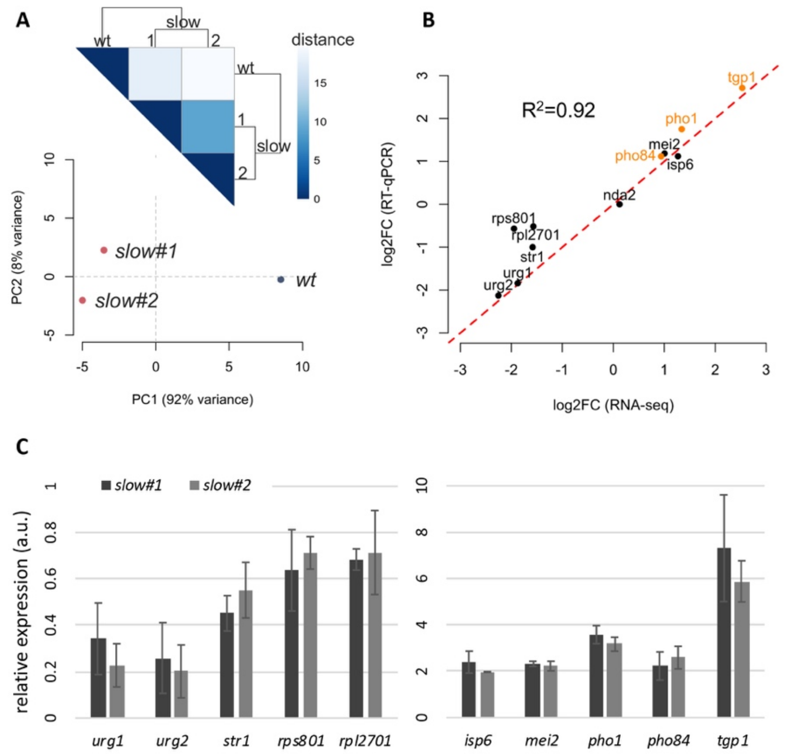

Supplemental Figure S3. **Validation of the RNA-seq data**

(A) *Top panel*: Hierarchical clustering based on the Euclidian distance between the *rlog-transformed* read counts in the slow mutants and wild-type strain. *Bottom panel*: principal component (PC) analysis of the *rlog-transformed* read counts in the slow mutants and wild-type strain.
(B) Correlation between the log2 fold change (FC) of expression of the slow mutant as measured by RNA-seq and RT-qPCR. Identity (y=x) is indicated as a dashed red line. Detailed RT-qPCR measurement of relative expression are shown in Fig. S3C below.
(C) Gene expression changes in the slow mutant relative to the wild-type using *nda2* as internal control for five down-regulated and five up-regulated genes (left and right panels, respectively). Error bars represent the standard deviation of the mean from three independent experiments.
