# Supplementary material for: Nutrient-dependent control of RNA polymerase II elongation rate regulates specific gene expression programs by alternative polyadenylation
Source: Genes Dev. 2020 Jul 1;34(13-14):883–97. doi: 10.1101/gad.337212.120 (PMC7328516; doi:10.1101/gad.337212.120)
Supplement: Supplemental Material [file supp_gad.337212.120_Supplemental_FigS4.pdf]

A) DOWN-REGULATED (n=293)	#genes	fold enrich.	FDR
**transmembrane (TM) transport**	43	2.4	2.17E-05
→ organic acid TM transport	12	4.7	1.12E-03
→ amino acid TM transport	11	5.7	1.76E-04
**ribosome biogenesis**	43	2.1	6.55E-04
→ rRNA processing	27	2.3	7.47E-03
**cytoplasmic translation**	31	2.2	4.75E-03
**iron assimilation**	4	14.0	7.61E-03

B) UP-REGULATED (n=343)	#genes	fold enrich.	FDR
**reproduction**	45	1.9	3.30E-03
→ sexual reproduction	26	2.2	8.81E-03
→ conjugation with cellular fusion	18	2.7	8.34E-03
→ pheromone-dependent signal transduction involved in conjugation with cellular fusion	9	7.0	4.06E-04
**purine metabolic process**	18	2.9	4.19E-03
→ purine monophosphate metabolism	18	3.2	1.71E-03
→ purine monophosphate biosynth.	10	4.2	7.72E-03
→ purine triphosphate metabolism	13	3.9	2.52E-03
→ ATP biosynthesis	8	6.2	2.63E-03

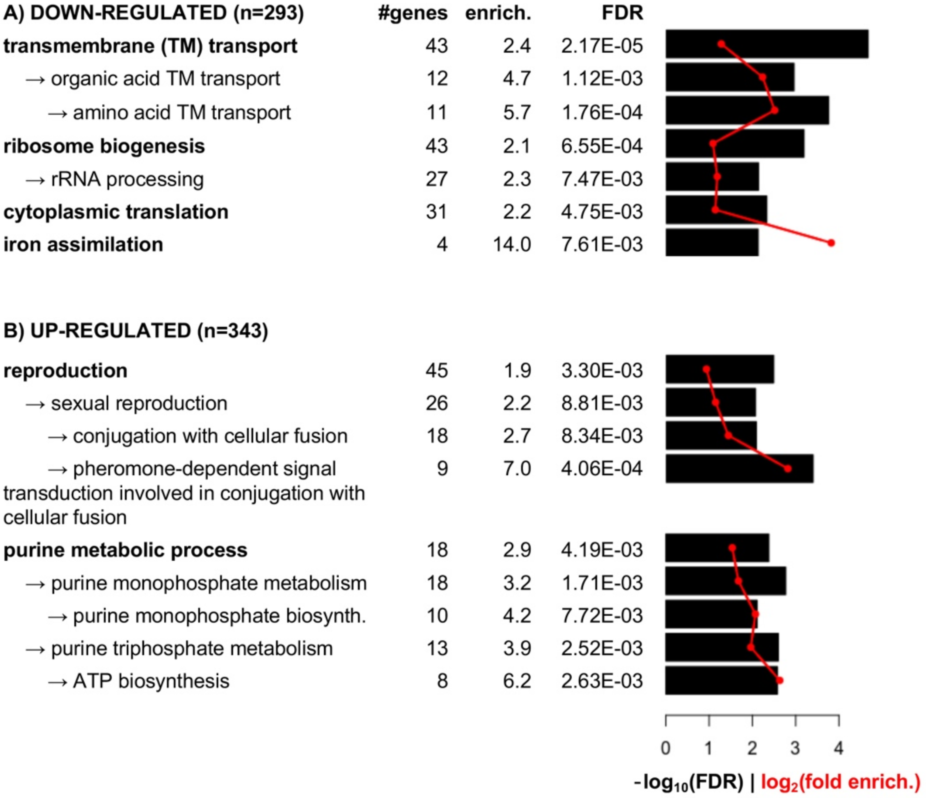

Supplemental Figure S4. **Functional analysis of differentially-expressed genes in *rpb1-N494D* cells.**

Angeli (Bitton et al. 2015) was used to compute the enrichment of gene ontology (GO) terms from the biological process branch of 293 and 343 protein-coding genes down- **(A)** or up-regulated **(B),** respectively, in the *slow* mutant with an absolute $\log_2$ fold-change > log2(1.5) and a FDR < 0.01. The significantly-enriched GO terms (FDR < 0.01) were organized according to their hierarchy after manual filtering against redundant terms. The number of up- or down-regulated genes in the category (*#genes*), the enrichment (*fold enrich.*), and the false discovery rate (*FDR*) are indicated in the table. The black-filled bars beside the table represent the significance of the enrichment expressed as the minus log of the FDR, while the red dots represent the strength of the enrichment expressed as the log of the fold-enrichment.
