# Supplementary material for: Nutrient-dependent control of RNA polymerase II elongation rate regulates specific gene expression programs by alternative polyadenylation
Source: Genes Dev. 2020 Jul 1;34(13-14):883–97. doi: 10.1101/gad.337212.120 (PMC7328516; doi:10.1101/gad.337212.120)
Supplement: Supplemental Material [file supp_gad.337212.120_Supplemental_FigS5.pdf]

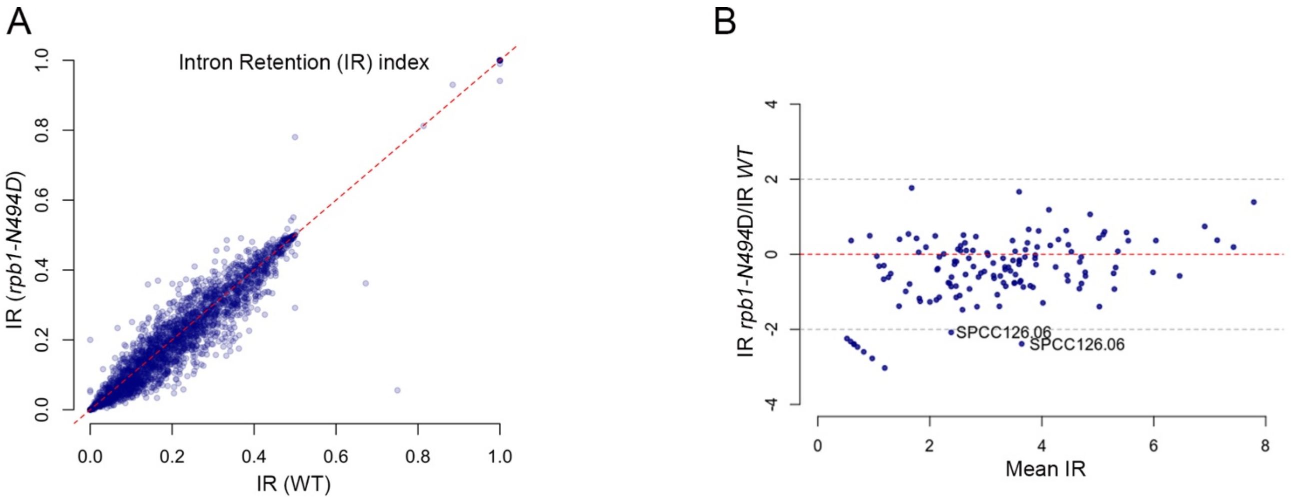

Supplemental Figure S5. ***rpb1-N494D* cells do not show a global splicing defect.**

(A) An “intron retention (IR) index”, defined as the proportion of intronic reads out of the sum of intronic reads and spliced reads (reads supporting exon-exon junctions), was calculated for all fission yeast genes with introns (n=2553 genes). Comparing the RNAPII slow mutant and the wild-type, the IR index globally distributes along the identity line (red dotted line), indicating the absence of a strong global effect of the *rpb1* slow mutation on splicing efficiency.

(B) The recently developed iREAD tool (Li et al. 2020) was applied to our RNA-seq data. iREAD calculates the number of intron retention events from total mapped reads. iREAD only considers introns that do not overlap any exons and that are supported by at least 1 intron-exon read, at least 20 total intronic reads (accounting for at least 3 FPKM), and with a “flatness score” of at least 0.9. 142 individual introns from 66 different genes met those stringent criteria in at least one of our conditions. The distribution of the log2 ratio of the iREAD FPKM score between the *rpb1* slow mutant and the wild-type strain does not support any strong global trend. Manual inspection of the identified introns revealed a single gene (*SPCC126.06*) with significantly less intron retention in the slow mutant.
