# Supplementary material for: Nutrient-dependent control of RNA polymerase II elongation rate regulates specific gene expression programs by alternative polyadenylation
Source: Genes Dev. 2020 Jul 1;34(13-14):883–97. doi: 10.1101/gad.337212.120 (PMC7328516; doi:10.1101/gad.337212.120)
Supplement: Supplemental Material [file supp_gad.337212.120_Supplemental_FigS6.pdf]

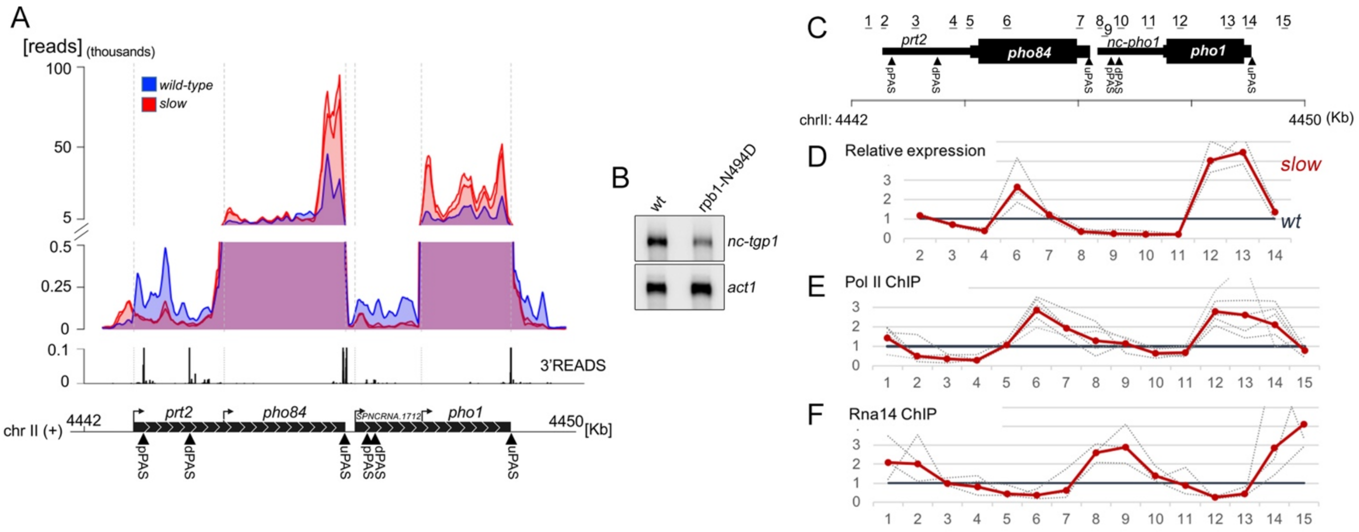

Supplemental Figure S6. **Reduced accumulation of upstream interfering lncRNAs at *pho84* and *pho1* loci in the RNAPII slow mutant**.

(A) Normalized read coverage (left axes) on the *prt2/pho84 and SPNCRNA.1712 (nc-pho1)/pho1* loci for the wild-type (blue area) and slow mutants (red areas) based on the RNA-seq data, and for previously published 3' READS data (Liu et al. 2017b) in wild-type cells (black peaks). Proximal (p), distal (d), and unique (u) PASs are indicated at the bottom.

(B) Analysis of *nc-tgp1* lncRNA level by RNase protection assay (RPA). Total RNA prepared from wild-type and *rpb1-N494D* cells was analyzed by RPA using specific riboprobes complementary to the *nc-tgp1* lncRNA (top panel) and to the *act1* mRNA (bottom panel).

(C) On-scale representation of the *prt2/pho84* and *nc-pho1/pho1* loci indicating the position of the amplicons (1-15) used for qRT-PCR and ChIP analyses as well as the position of the proximal (pPAS), distal (dPAS), and unique (uPAS) polyA sites.

(D) RT-qPCR analysis of *prt2/pho84* and *nc-pho1/pho1* expression relative to wild-type in three independent experiments. The blue and red lines indicate the baseline wild-type level and the mean for the slow profiles, respectively. The individual replicates are shown as dashed lines.

(E) Relative RNAPII occupancy in the slow mutant compared to the wild-type at the *prt2/pho84* and *nc-pho1/pho1* loci from nine independent experiments. The blue and red lines correspond to the baseline wild-type level and the mean value for the slow mutant, respectively. The individual replicates are shown as dashed lines.

(F) Chromatin occupancy of Rna14 at the *prt2/pho84* and *nc-pho1/pho1* loci in the RNAPII slow mutant (red line) relative to the wild-type control (blue line). Individual replicates (n=3; dashed lines) were normalized to total RNAPII levels measured from the same chromatin preparation at each region and expressed relative to the wild-type.
