# Supplementary material for: Nutrient-dependent control of RNA polymerase II elongation rate regulates specific gene expression programs by alternative polyadenylation
Source: Genes Dev. 2020 Jul 1;34(13-14):883–97. doi: 10.1101/gad.337212.120 (PMC7328516; doi:10.1101/gad.337212.120)
Supplement: Supplemental Material [file supp_gad.337212.120_Supplemental_FigS7.pdf]

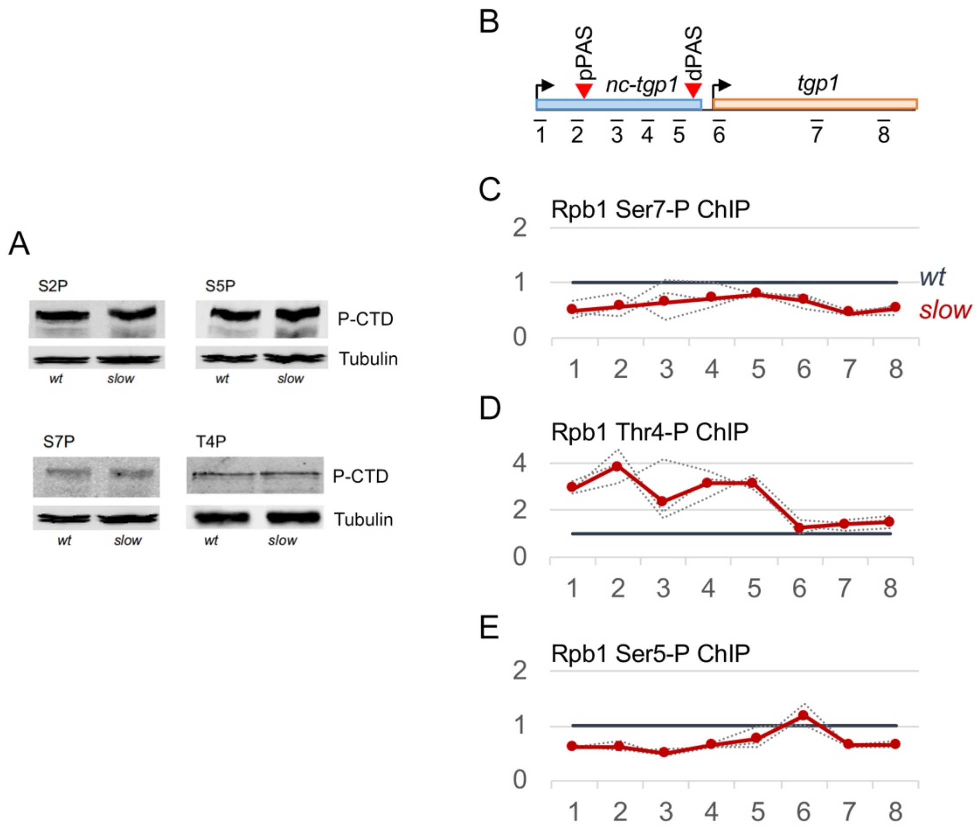

Supplemental Figure S7. **Distribution of Ser7, Thr4, and Ser5 CTD phosphorylation at the *nc-tgp1*/*tgp1* locus in the RNAPII slow mutant.**

(A) Western blot analysis of the indicated CTD phosphorylation (P-CTD) marks using total extracts prepared from the wild-type (wt) strain and the *rpb1* slow mutant.
(B) On-scale representation of the *nc-tgp1/tgp1* locus indicating the positions of the amplicons (1-8) used for ChIP analysis of CTD modifications as well as the two polyA sites (PAS) in *nc-tgp1.*
(C-E) Relative chromatin occupancy of Rpb1 CTD Ser7 **(C)**, Thr4 **(D)**, and Ser5 **(E)** phosphorylation at the *nc-tgp1/tgp1* locus in the slow mutant (red line) relative to the wild-type control (blue line). The individual replicates (n=3, dashed lines) were normalized to total RNAPII levels measured from the same chromatin preparation and expressed relative to the wild-type strain (blue line).
