# Supplementary material for: Nutrient-dependent control of RNA polymerase II elongation rate regulates specific gene expression programs by alternative polyadenylation
Source: Genes Dev. 2020 Jul 1;34(13-14):883–97. doi: 10.1101/gad.337212.120 (PMC7328516; doi:10.1101/gad.337212.120)
Supplement: Supplemental Material [file supp_gad.337212.120_Supplemental_FigS8.pdf]

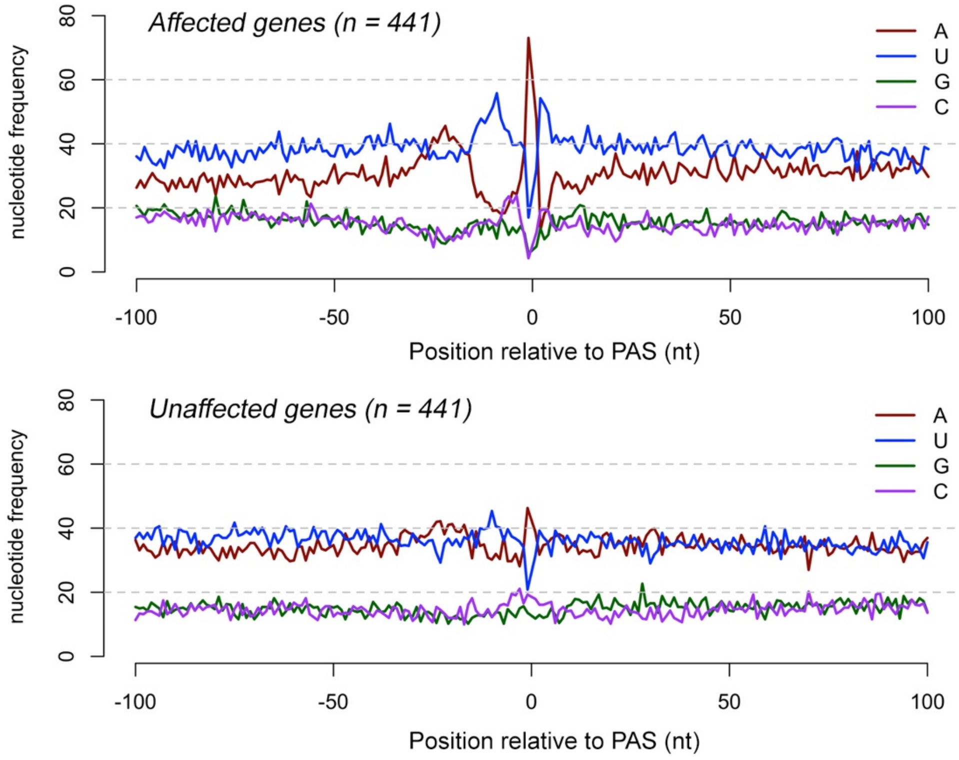

Supplemental Figure S8. **Nucleotide frequencies surrounding the proximal PASs of affected (*top*) and unaffected (*bottom*) genes in the RNAPII slow mutant**.
